# Fibroblasts enhance the growth and survival of adult feline small intestinal organoids

**DOI:** 10.1128/msphere.00290-25

**Published:** 2025-08-08

**Authors:** Nicole D. Hryckowian, Katelyn Studener, Waneska S. Frizzarini, David Arranz-Solís, Roberto Sánchez-Sánchez, Laura J. Knoll

**Affiliations:** 1 Department of Medical Microbiology & Immunology, University of Wisconsin-Madison, Madison, Wisconsin, USA; 2 SALUVET, Animal Health Department, Faculty of Veterinary Sciences, Complutense University of Madrid, Madrid, Spain; Virginia-Maryland College of Veterinary Medicine, Blacksburg, Virginia, USA

**Keywords:** *Toxoplasma gondii *, enteroid, organoid, small intestine, fibroblast, feline

## Abstract

Intestinal organoids are important cell culture models that complement live animal studies of many intestinal pathogens. Adult feline small intestinal organoids are needed for infectious disease research but are difficult to work with due to slow growth and premature senescence. We introduce a method of co-culturing adult feline small intestinal organoids with growth-inhibited human foreskin fibroblast feeder cells to enhance organoid proliferation and survival. With feeder cells, feline jejunal and ileal organoids survived at least 9 months in culture until cryopreservation. Fibroblast supplementation increased the maximum size of cat and mouse intestinal organoids as well. The increased longevity and size of these organoids are a significant improvement on current methods. These organoids also supported pre-sexual development of the medically important parasite *Toxoplasma gondii*, as evidenced by expression of the merozoite-specific marker GRA11b. This GRA11b positivity was higher in mature cat organoid-derived monolayers grown for 21 days prior to infection, compared with monolayers grown for 10 days. These methods have high potential to reduce the number of cats used for infectious disease research and may be applicable for intestinal cells from other animals that are difficult to culture.

**IMPORTANCE:**

Many microbial pathogens are acquired orally through contaminated food or water. Being able to model these infections in cell culture has been greatly enhanced by the development of intestinal organoid technology. One of the species that hosts several infections is cats, but cat intestinal organoids have been notoriously difficult to grow. Here, we describe a co-culture method with fibroblast cells that dramatically improves the longevity of adult cat intestinal organoids. These cat organoid cells can support the pre-sexual development stages of the intestinal pathogen *Toxoplasma gondii*, a parasite whose sexual cycle is restricted to cats and is the reason that pregnant women are told not to change the litter box. These culture conditions will be a resource to study other cat intestinal pathogens and intestinal organoids from other animals that are difficult to culture.

## INTRODUCTION

The small intestinal epithelium is a single layer of columnar epithelial cells that provide barrier function and absorb nutrients. The epithelium is a crucial component of host defense to orally acquired enteric pathogens like the single-celled eukaryotic parasite *Toxoplasma gondii*. Approximately 30% of humans are chronically infected with *T. gondii* (1), and most infections are acquired orally. Oral infection is also key for the *T. gondii* life cycle when it occurs in the definitive feline host. The feline small intestinal epithelium is the only site where parasites undergo sexual reproduction to produce highly infectious and environmentally resistant oocysts. Understanding how *T. gondii* behaves in the cat intestine is crucial to interrupting transmission, but using live cats in *T. gondii* research is fraught with ethical concerns.

**Fig 1:**
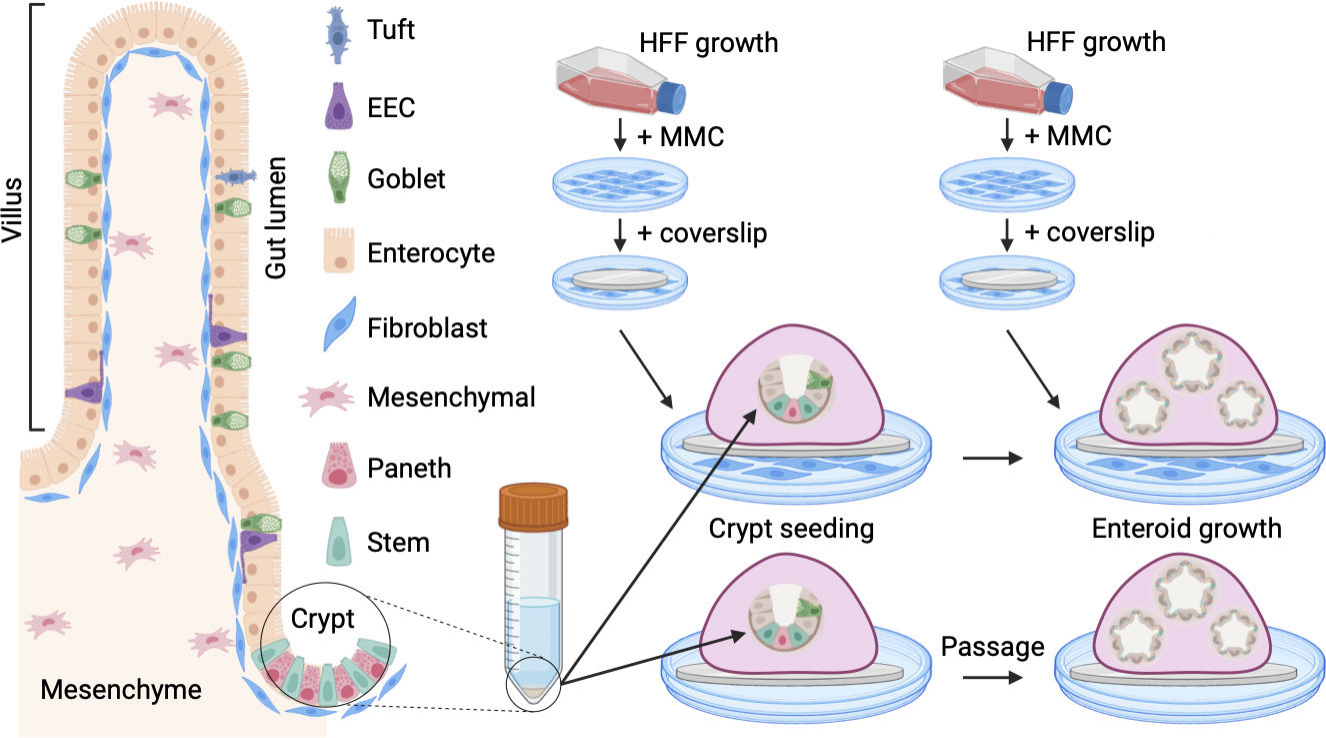
Small intestinal anatomy and fibroblast supplementation of enteroids. A schematic of healthy small intestinal epithelium is shown at left, along with the common cell types of the small intestine and gut compartments discussed in this paper. EEC = enteroendocrine. HFF = human foreskin fibroblast. MMC = mitomycin C. A simplified schematic of crypt isolation for enteroid preparation with and without HFFs is shown at right. New HFFs must be prepared prior to each enteroid passage. Image created with Biorender.com.

An alternative to studying *T. gondii* in live cats is feline intestinal organoids (enteroids). Enteroids are derived from intestinal crypt stem cells that divide and differentiate into 3D structures that mimic the cell types and architecture of the intestinal epithelium (Fig. 1). Mouse and human enteroids have enabled important discoveries in infectious disease research, including studies of Apicomplexan parasites (2–4). Although feline enteroids have been used for infectious disease research by our lab and others, they remain difficult to work with because they undergo early growth arrest and cell death (5, 6).

*In vivo*, intestinal stem cells rely on growth factors from crypt-resident Paneth cells and underlying mesenchymal cells to maintain stemness and proliferative capacity (7, 8). A major advancement in organoid technology was the discovery that the mesenchymal niche was not required for stem cell maintenance as long as the appropriate growth factors were provided in enteroid media (6, 7). Many species have been successfully cultivated under these conditions, including human, mouse, dog, cow, sheep, horse, chicken, and pig enteroids (5, 6). The slow growth and early senescence of feline enteroids suggested to us and others (5, 6) that they might require growth factors not provided in traditional enteroid media. Tekes et al. (6) used a human enteroid medium formulation, which delayed but did not rescue feline enteroid death. Powell & Behnke (5) tested many additional growth factors for their ability to support cat enteroids, including FGF-2,-4, and -10, nicotinamide, IGF-1, PGE2, and Wnt-2b and Gremlin, but none rescued early growth arrest.

The same study saw that mesenchymal marker vimentin (VIM) + mesenchyme-like cells were present in early cat enteroid passages. When these mesenchyme-like cells disappeared around passage 10, the cat enteroids began to decline. This suggested that mesenchymal cells provide necessary growth factors for the long-term propagation of cat enteroids. We tested this idea by supplementing adult cat jejunal and ileal enteroids with mitomycin-C inactivated human foreskin fibroblasts (HFFs). We found that these HFF feeder cells were sufficient to support the long-term growth of small intestinal jejunum enteroids and some ileum enteroids. HFF-supplemented small intestinal enteroids from cats and some mouse samples were also larger than non-supplemented enteroids, which may facilitate easier microinjection.

We also found that fibroblast-supplemented cat enteroid monolayers supported pre-sexual development of *T. gondii*, particularly in monolayers grown for 3–4 weeks prior to infection. Given the longevity of these adult cat enteroids and their permissiveness to *T. gondii* sexual development, we expect these organoids will be useful to study *Toxoplasma* and other feline infectious diseases. Beyond felines, the application of our methods may provide similar benefits to other organoid-dependent studies.

## RESULTS

### Selection of organoid medium formulation

Our laboratory previously grew fetal small intestinal organoids from the domestic cat (*Felis catus*) to study host–parasite interactions in *T. gondii* infection (9). However, the enteroids grew slowly and underwent growth arrest and cell death after 2–5 passages in culture. To determine whether we could optimize our media for cat enteroid growth, we consulted previous studies that used enteroids for infectious disease research (Table 1) (5, 6, 10). The human organoid medium components nicotinamide, A-83-01, SB202190, and gastrin supported feline organoid growth for 10 additional passages in a previous study (6). Powell & Behnke (5) tested additional stem cell-promoting compounds, but they did not extend organoid survival. As such, we added A-83-01, SB202190, and gastrin to our original medium formula (9).

**TABLE 1:** Organoid medium formulations that enable cat enteroid growth^*a*^^,^^*b*^

Reagent	Final concentration	Co et al.	Powell and Behnke	Tekes et al.	Martorelli di Genova	This study
L-WRN conditioned media	50%	x	x	x	x	x
Adv. DMEM/F12	50%	x		x	x	x
Penicillin/streptomycin	1%			x	x	x
HEPES	1-20 mM	1 mM		10 mM	20 mM	10 mM
GlutaMAX	2 mM (1 x)	x		x	x	x
B27	1x	x			x	x
N-2	1x				x	x
N-acetylcysteine	1 mM	x		x		x
Nicotinamide	10 mM	x		x	x	x
EGF	50 ng/mL	x		x	x	x
A83-01	0.5 µM	x		x		x
SB202190	0.5-10 µM	10 µM		0.5 µM		10 µM
Y-27632	10 µM	x		x	x	x
CHIR99021	0.25-3 µM	0.25 µM			3 µM	2.5 µM
ITS	1x				x	x
Gastrin	10 nM	x		x		x
Matrigel	75-100%		x	x	x	x
Cultrex	75-100%	x				
						
Additional growth factors attempted in conjunction with L-WRN conditioned media (Powell & Behnke)						
FGF-2	Not specified		x			
FGF-4	Not specified		x			
FGF-10 + Nicotinamide	Not specified		x			
IGF-1	Not specified		x			
PGE2	Not specified		x			
Wnt-2b + Gremlin	Not specified		x			
A83−01 + SB202190	Not specified		x			
Max cat passage no.		Not specified	21	15	Not specified	45
Max days in culture		Not specified	67	Not specified	Not specified	288

^*a*^ To develop an optimal growth media for feline enteroids, we consulted medium formulations from previous studies that utilized human (10), cat (6), and a suite of species including cat, dog, cow, horse, sheep, pig, and chicken (5). Components used in each study, including the present study, are shown along with their maximum cat enteroid survival times. Tekes et al. list several medium formulations in their study; we list only their human medium formulation, which was most successful for cat enteroid culture. ITS = insulin transferrin selenium supplement.

^*b*^ x indicates that the amount indicated under “Final concentration” was used.

### Human foreskin fibroblasts enable long-term passage and increase the size of feline enteroids

Powell & Behnke (5) noted that feline enteroids declined after the loss of VIM + mesenchymal-like cells in their cultures. VIM is a marker for mesenchymal cells like fibroblasts, which support adult stem cells *in vivo* and in some models of intestinal cell culture, such as induced pluripotent stem cells (iPSCs) and organoid-derived monolayers (8, 11–13). We tested whether HFFs co-cultured with feline enteroids would enable their long-term survival. We chose HFFs because they are low maintenance, commercially available, and commonly used to culture *T. gondii*. To inactivate fibroblasts, we used mitomycin C (MMC), a double-stranded DNA alkylating agent that is used to growth-inactivate mouse embryonic fibroblasts for mouse embryonic stem cell culture (14). After 2 h of MMC treatment, HFFs were trypsinized and seeded into 24-well plates for enteroid seeding the next day (Fig. 1).

**Fig 2:**
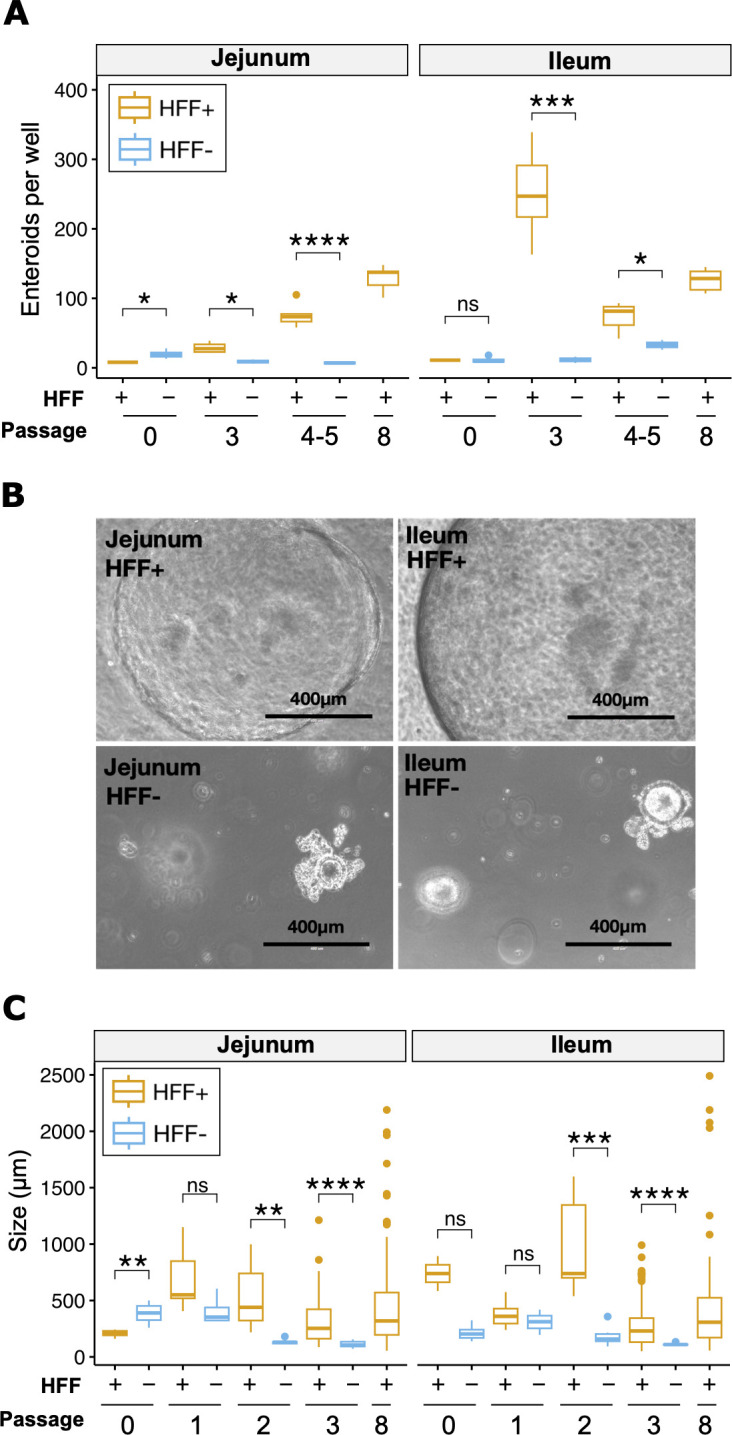
Fibroblasts promote early outgrowth of feline jejunum and ileum enteroids. Cat 0 enteroids were monitored from passage 0 to passage 8 to obtain enteroid counts per well (**A**), photos of passage 2 enteroids at 10× magnification using the EVOS imaging system (**B**), and enteroid diameters (**C**). For diameters, at least five organoids per well were selected at random. If wells contained fewer than five organoids, all were measured. All available wells were monitored, with at least two wells per condition, until non-supplemented enteroids began to die, at which point only one well remained. Organoids without fibroblasts died by passage 5, see also Table 2. *, *P* < 0.05; **, *P* < 0.01; ***, *P* < 0.001; ****, *P* < 0.0001, ns, not significant, Welch’s *t*-test.

To pilot our fibroblast protocol, we isolated jejunal and ileal crypts from one adult feline (cat 0), cultured them in Matrigel and organoid media, and passaged them continuously with or without HFFs (Fig. 1). Enteroids grew out of all crypt samples at 10–20 enteroids per well at passage 0 (Fig. 2A). By passage 3, however, HFF-supplemented enteroids were more numerous than unsupplemented enteroids. By passage 4, just one unsupplemented well of jejunal and ileal enteroids remained, with 10 and 40 enteroids, respectively. All remaining enteroids died by passage 5. Individual organoids were determined to be dead or dying when spheroids appeared dark brown versus golden under brightfield microscopy and when they extruded debris into the surrounding Matrigel. Organoid cultures overall were considered dead after 2–3 passages, when they failed to grow in size or increase in number. In contrast, HFF-supplemented enteroids from both tissues increased to 40–250 enteroids per well by passage 4. Enteroids from both tissues maintained their high proliferative capacity out to passage 8.

**TABLE 2:** Cat enteroid survival times^*a*^

Cat	Crypt isolation date	Tissue	Fibroblast treatment	Passage start or passage at fibroblast withdrawal	Passage end	Date start	Date end	Days in culture	Reason for termination	Notes
Samples continuously passed										
0	2/27/23	Jejunum	HFF	0	45	2/27/23	12/12/23	288	Froze	
0	2/27/23	Ileum	HFF	0	45	2/27/23	12/12/23	288	Froze	
0	2/27/23	Jejunum	None	0	5	2/27/23	4/17/23	49	Died	
0	2/27/23	Ileum	None	0	5	2/27/23	4/17/23	49	Died	
1	6/14/23	Jejunum	HFF	0	29	6/14/23	12/12/23	181	Froze	
1	6/14/23	Ileum	HFF	0	7	6/22/23	7/29/23	37	Died	
1	6/14/23	Jejunum	None	0	11	6/14/23	8/24/23	71	Died	
1	6/14/23	Ileum	None	0	13	6/14/23	9/20/23	98	Died	
2	6/22/23	Jejunum	HFF	0	26	6/22/23	12/12/23	173	Froze	
2	6/22/23	Ileum	HFF	0	4	6/22/23	8/3/23	42	Died	
2	6/22/23	Jejunum	None	0	8	6/22/23	8/15/23	54	Died	
2	6/22/23	Ileum	None	0	5	6/22/23	7/26/23	34	Died	
3	7/12/23	Jejunum	HFF	0	23	7/12/23	12/12/23	153	Froze	
3	7/12/23	Ileum	HFF	0	9	7/12/23	9/14/23	64	Died	Died suddenly 9/14, culture had debris resembling fungal hyphae
Samples for fibroblast withdrawal experiments										
0	2/27/23	Ileum		12	23	5/18/23	8/15/23	89	Died	
0	2/27/23	Jejunum		17	44	6/27/23	12/12/23	168	Froze	
0	2/27/23	Ileum		17	44	6/27/23	12/12/23	168	Froze	This sample was the only one with a budding phenotype
3	7/12/23	Jejunum		4	22	8/8/23	12/12/23	126	Froze	
3	7/12/23	Ileum		4	22	8/8/23	12/12/23	126	Died	

^*a*^ Length of time in culture (passage number and total days) is listed for jejunum and ileum for each of the four cats used in this study. Samples used for fibroblast withdrawal experiments (cats 0 and 3) were offshoots of samples continuously passed with fibroblasts.

**Fig 3:**
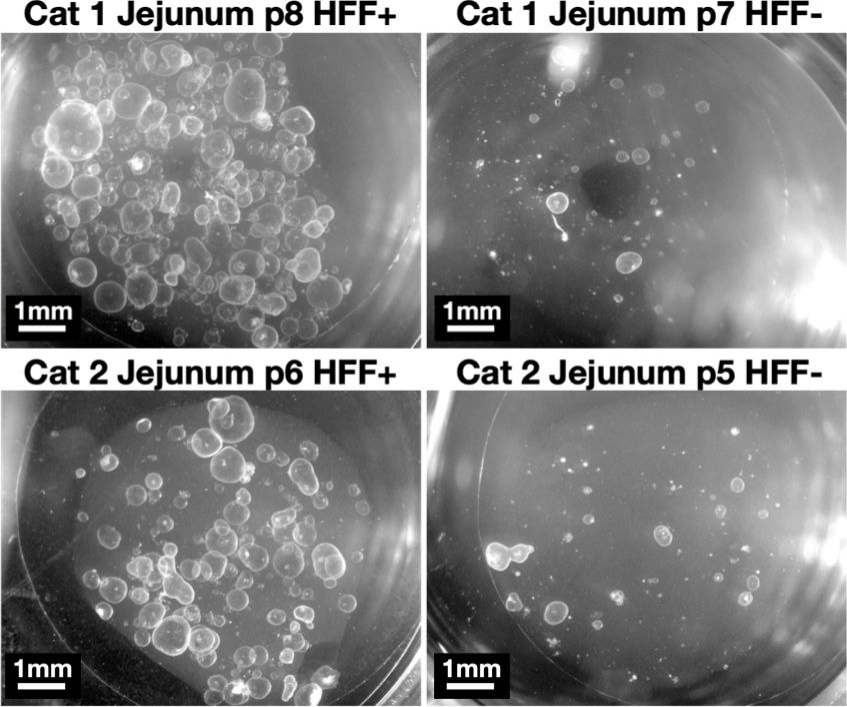
Fibroblasts increase the size and abundance of feline small intestinal jejunum enteroids. Representative photos of enteroids from cats 1 and 2 grown with (left) and without (right) HFF supplementation. All photos were collected on the same date using Zeiss Zen software and a stereo microscope at 4× magnification.

We used the same enteroids from cat 0 to determine whether fibroblasts increase enteroid size (Fig. 2B and C). At passage 0, enteroids ranged in diameter from ~200 to 700 µm, with no significant increase in size due to HFF supplementation for jejunum samples. HFF-supplemented ileum organoids were slightly larger, but this trend did not reach statistical significance, perhaps due to low sample size. Unsupplemented enteroids did not get any larger in subsequent passages, instead becoming smaller by passage 3 when they ceased proliferating. HFF-supplemented enteroids maintained mean diameters of at least 200 µm from passage to passage, with a small number of enteroids in these cultures consistently reaching 1–2 mm diameters. To test whether fibroblast feeder cells enhance enteroid survival in additional animals, we applied the HFF supplementation protocol to three other felines and tracked their survival (Table 2). All HFF-supplemented jejunal enteroids survived 126–288 days in culture (passage 23–45), at which point they were preserved in liquid nitrogen. Ileal tissue was more variable. The ileal sample from cat 0 survived long-term, but the samples from cats 1–3 died between passages 4 and 9. Although the ileal enteroids from cat 3 showed no signs of early senescence, they became contaminated with fungi at passage 9 and died shortly afterward. HFF-supplemented enteroids were more abundant and larger than unsupplemented enteroids (Fig. 3), including early ileum passages from cats 1 to 3.

### Murine enteroids are less responsive than feline enteroids to fibroblast supplementation

**Fig 4:**
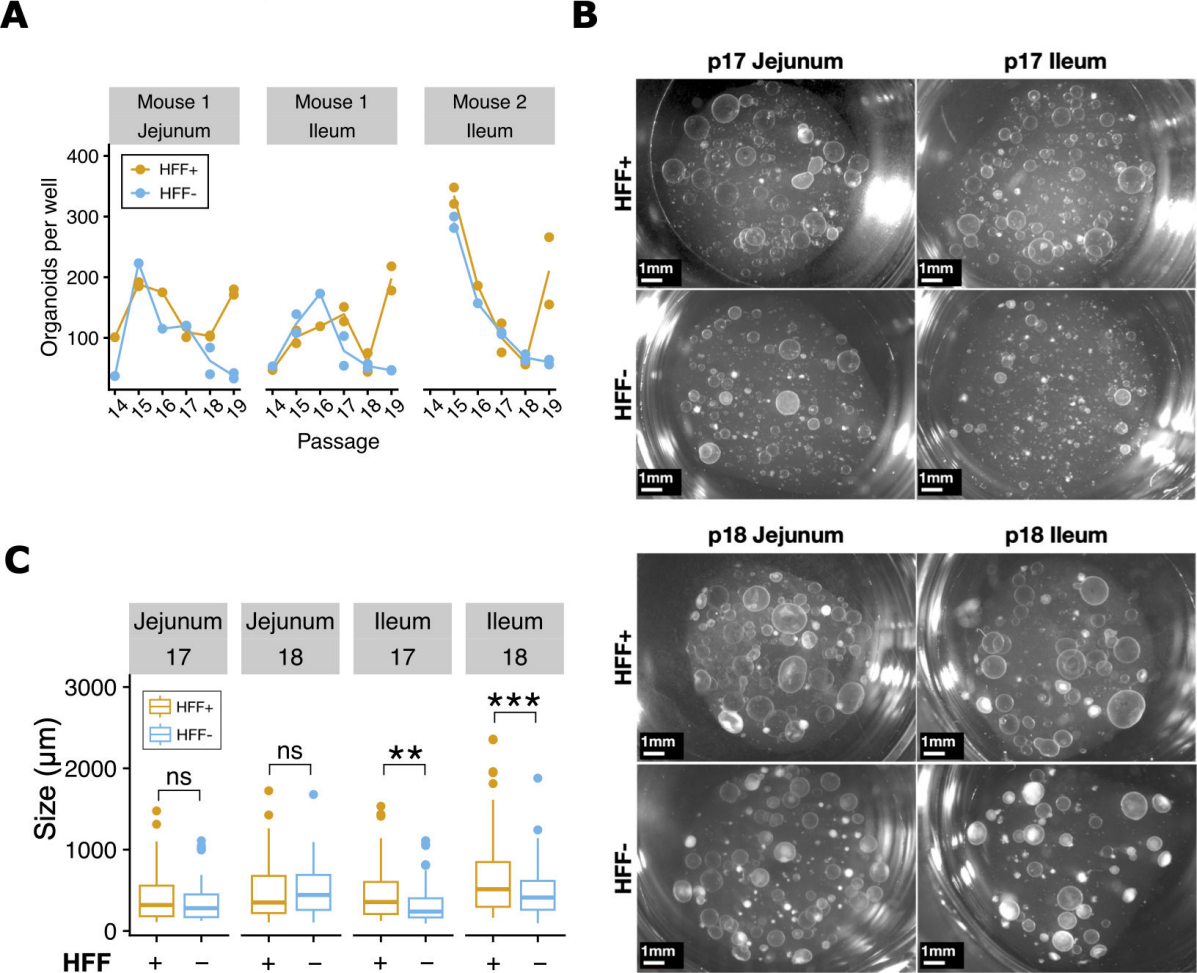
Murine enteroids are less responsive to fibroblasts than feline enteroids. (**A**) Per-well enumeration of murine jejunum (*n* = 1 animal) and ileum (*n* = 2 animals) enteroids throughout 6 passages of continuous maintenance with or without fibroblasts (*n* = 1-2 wells per condition). No statistically significant differences were detected by a paired *t*-test. (**B**) Representative phase images of enteroids from mouse 1 jejunum and ileum with and without HFF supplementation at passages 17 and 18; 4× magnification, scalebar = 1 mm. (**C**) The diameter of enteroids (*n* = 25 per well, *n* = 2 wells per condition for jejunum and *n* = 4 wells per condition for ileum) was measured just before passaging for two consecutive passages, 17 and 18. **, *P* < 0.01; ***, *P* < 0.0001, ns, not significant, Welch’s *t*-test.

Murine enteroids do not require fibroblast feeder cells for survival, but we reasoned that they might respond to growth-enhancing signals from fibroblasts. To test whether HFF feeder cells would increase murine enteroid size or abundance (Fig. 4), we continuously passaged murine enteroids with HFFs from passages 14 to 19. HFF supplementation occasionally but not always increased enteroid counts in mouse jejunum and ileum samples (Fig. 4A). At passage 19, HFF-supplemented organoids were more abundant than their unsupplemented controls. We did not collect data beyond passage 19; hence, it remains unknown whether that increase in abundance would have persisted in future passages. We did notice a significant increase in size for ileum enteroids (Fig. 4B), whereas jejunum enteroid size was less responsive to HFF supplementation. Overall, mouse and cat enteroids reached similar maximum sizes of 1–2 mm (Fig. 2C and 4C) and abundances of 200–300 enteroids per well (Fig. 2A), with similar spherical (cystic) phenotypes (Fig. 3 and 4B).

### Long-term enteroid survival is possible following fibroblast withdrawal

**Fig 5:**
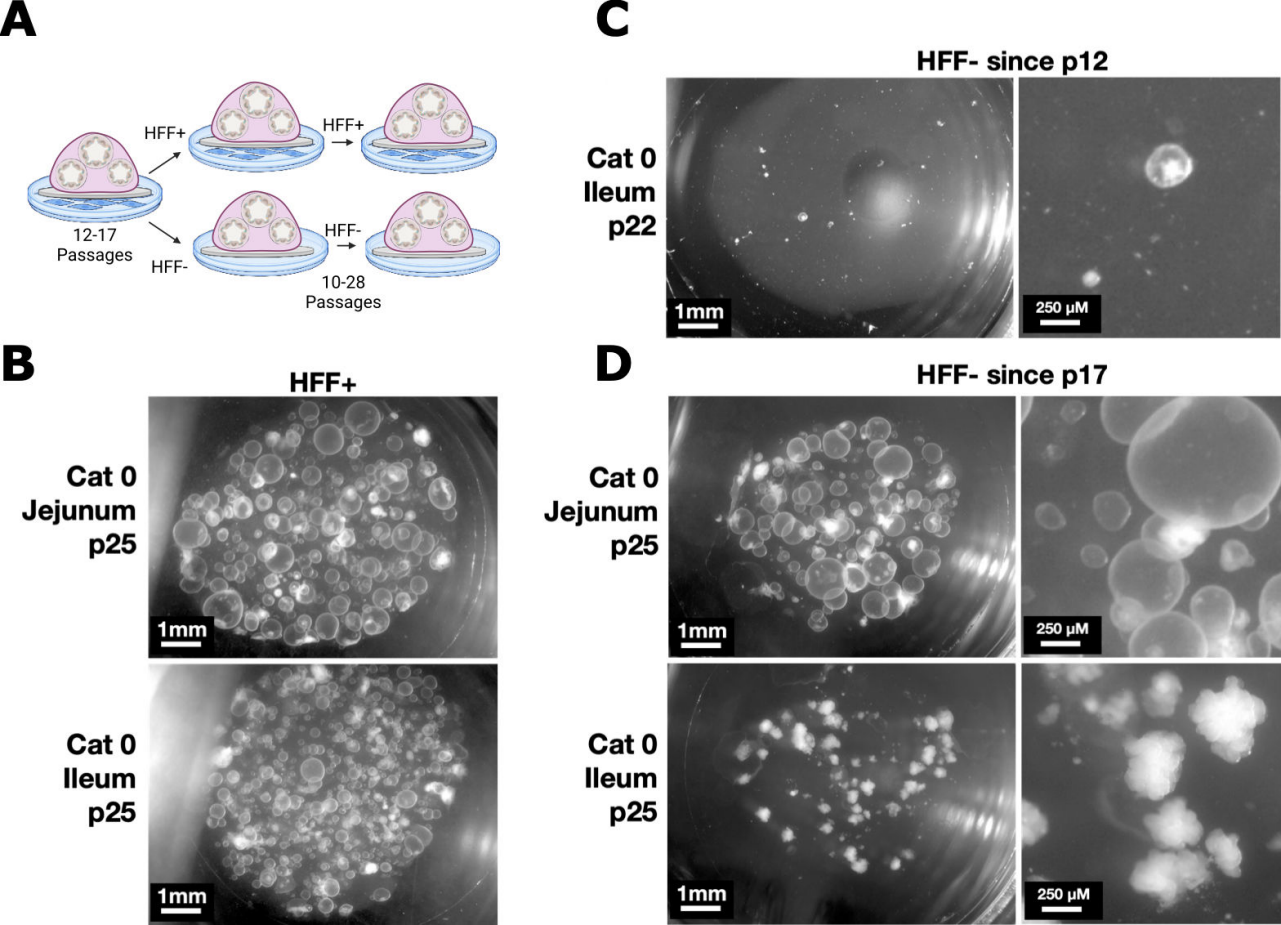
Long-term feline enteroid survival is possible following fibroblast withdrawal. (**A**) Schematic of experimental set-up. Cat 0 jejunum and ileum samples were initially grown on fibroblasts until fibroblast withdrawal at the indicated passage number, either 12 or 17. They were then split into two treatments: either continued fibroblast supplementation or continuous passage without fibroblast supplementation. Image created with Biorender.com. (**B**) Representative images of passage 25 cat 0 jejunum and ileum enteroids continuously passaged with HFFs. (**C**) Representative images of passage 22 cat 0 ileum enteroids that were grown without HFFs since passage 12. (**D**) Representative images of passage 25 cat jejunum and ileum enteroids that were grown without HFFs since passage 17. Note the budding phenotype of the ileum sample.

Because fibroblast feeder cells require additional time and resources to maintain, we next tested whether we could stop fibroblast supplementation after enteroids were well-established (Table 2; Fig. 5A). We used samples from cats 0 and 3 for these experiments based on tissue availability and compared unsupplemented samples with their parent samples that were continuously cultured with fibroblasts (Fig. 5B). For cat 3 (Table 2), we removed jejunum and ileum samples from fibroblasts at passage 4. Ileum enteroids remained abundant for around 15 passages but then dwindled until all were gone at day 126/passage 22. The jejunum enteroids appeared healthy through day 126/passage 22, at which point we preserved them in liquid nitrogen. An ileum sample from cat 0 was removed from fibroblasts at passage 12 and survived an additional 89 days/11 passages without fibroblast supplementation before enteroid death (Table 2). Enteroids were increasingly small and sparse leading up to their death (Fig. 5C). To test whether removing enteroids from fibroblasts at a later passage would enable longer survival, cat 0 jejunum and ileum were removed from fibroblasts at passage 17. Unlike the enteroids removed from fibroblasts at passage 12, both the jejunum and ileum enteroids survived at high abundance for an additional 168 days/27 passages prior to liquid nitrogen preservation (Table 2; Fig. 5D). Although we did not systematically test whether organoids were viable after a freeze-thaw, the few samples we tested were still viable after liquid nitrogen preservation.

### Cat enteroid-derived monolayers support *T. gondii* sexual development studies

**Fig 6:**
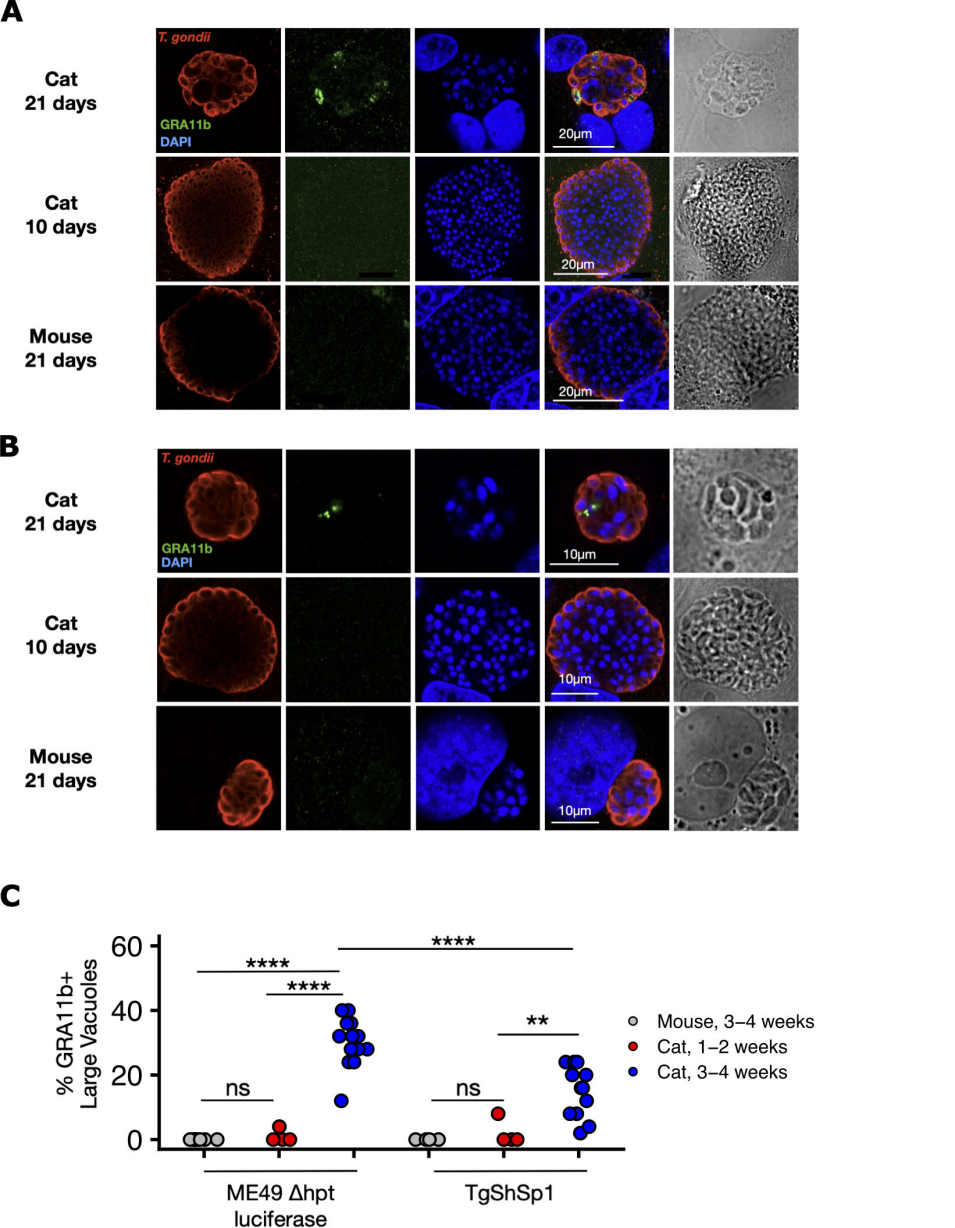
Mature feline enteroid monolayers support *T. gondii* pre-sexual development. (**A**) Cat or mouse enteroid-derived monolayers were grown for 1–4 weeks and infected with 20,000 mouse brain-derived *T. gondii* bradyzoites, Type II strain ME49 ∆hpt luciferase. After 5 days of infection, monolayers were fixed, stained with antibodies against *T. gondii* (red) and against the pre-sexual *T. gondii* protein GRA11b (green), counterstained with DAPI (blue), and imaged by confocal microscopy. (**B**) Similar to (**A**) but with *T. gondii* Type II strain TgShSp1. (**C**) Quantification of GRA11b-positive vacuoles from (**A**) and (**B**). At least 25 large (>=8 parasites for TgShSp1, >16 parasites for ME49 ∆hpt luciferase due to faster growth) vacuoles were counted from each coverslip. At least four replicate coverslips were counted for each condition and parasite strain. **, *P* < 0.01; ****, *P* < 0.0001, ns, not significant, Welch’s *t*-test.

Our lab previously used fetal cat enteroids for studying cat-specific life stages of the Apicomplexan parasite *T. gondii* (9). Unfortunately, fetal organoids ceased to expand around passage 5, which limited their utility. To test whether our long-lived adult enteroids supported *T. gondii* sexual development, we grew cat- and mouse-derived enteroids as monolayers for 1–4 weeks. One day prior to infection, we switched the organoids to a modified organoid medium we term “infection” medium (see Materials and Methods). On infection day, we infected the ODMs with mouse brain-derived bradyzoites from two Type II strains of *T. gondii*, ME49 ∆hpt luciferase and TgShSp1. After 5 days of infection, we fixed the infected monolayers and stained with a polyclonal *T. gondii* antibody and a monoclonal antibody against GRA11b, a dense granule protein that serves as a marker for pre-sexual stages of *T. gondii* (9, 15, 16) (Fig. 6). Although TgShSp1 grew more slowly than ME49 ∆hpt luciferase, both parasites grew well in all monolayers, as evidenced by large vacuoles and some host cell lysis. As expected, we saw no GRA11b expression in mouse enteroids. Cat enteroids supported GRA11b expression in both *T. gondii* strains, although staining was usually restricted to just a few parasites in larger vacuoles. GRA11b positivity was higher in mature cat ODMs grown for 3–4 weeks prior to infection, and higher overall in the ME49 ∆hpt luciferase strain (Fig. 6C).

## DISCUSSION

We showed that feline small intestinal enteroids survive much longer than those in previous studies (5, 6) if they are supplemented with growth-inactivated HFFs. Jejunal enteroids were especially responsive to HFF supplementation, with a survival rate of 100% (4/4 animals). Ileal enteroids were less responsive, with just one-third of uncontaminated cultures surviving (see Table 2 for organoid lifespans and reasons for discontinuation in the study). Ileum enteroids tend to be less proliferative than enteroids from higher in the gastrointestinal tract, which may warrant higher crypt seeding densities, more frequent changes of media and HFFs, or perhaps feline intestinal mesenchymal fibroblasts rather than fibroblasts from human foreskin. Mouse enteroids were less responsive to HFF feeder cells, although ileum enteroids were larger following HFF supplementation. This finding is consistent with previous findings (7) that mouse enteroids do not need fibroblasts for long-term growth and survival.

It remains unclear why cats are uniquely reliant on fibroblast supplementation for enteroid survival. A past study found that chemically fixed and freeze-dried feeder cells were sufficient for supporting inducible pluripotent stem cells (iPSCs), suggesting that feeder cell protein(s) promoted stem cell attachment and survival through direct cell-to-cell contact (17). Feeder cells likely operate differently in our system because we prevent direct contact between feeder cells and organoids with a glass coverslip. Fibroblasts may secrete a needed growth factor that is not included in standard organoid media (5, 18). Cats may also lack stem cell-supportive Paneth cells (19) or have them at lower abundance than other species (6), making them more reliant on other cell types for needed growth factors. Paneth cell ablation in mice results in enteroid death unless Paneth cell products like Wnt3a are provided in the media or by other epithelial or mesenchymal cells (20–22). Finally, it is possible that the growth factors in standard organoid media are not optimized for cats or for the small intestine. For example, replacing the standard organoid medium component epidermal growth factor (EGF) with a similar but intestine-specific growth factor epiregulin results in more physiologically relevant human enteroid cultures (23, 24). Addition of other species- or tissue-specific growth factors is sometimes required to maintain gastrointestinal organoids (6, 10). Identifying the growth factors required for cat enteroid growth will be an interesting area of future study.

Previous work in our lab (9) used fetal feline organoids at passage 5 or lower to induce GRA11b expression in *T. gondii*. In that study, 30%–35% of parasite vacuoles were GRA11b-positive, and most parasites in a given vacuole were GRA11b-positive. In contrast, we saw 20%–30% GRA11b positivity in the ME49 and TgShSp1 parasite strains we tested in this study, with just a handful of parasites within a vacuole staining positive. We also saw that GRA11b remained inside parasites in this study, whereas in Martorelli di Genova et al., GRA11b was often observed outside of parasites in the parasitophorous vacuole. Finally, we excluded small vacuoles from analysis, as they were rarely GRA11b-positive and would have biased toward lysis and reinvasion events. The Martorelli di Genova study did not exclude small vacuoles. Ultimately, this means that we saw less GRA11b positivity with our new organoid model.

We anticipate that GRA11b is less abundant due to host factors that differ between low-passage fetal organoids (9) and medium-to-high passage adult organoids (this study). We are currently testing the hypothesis that GRA11b is low because adult organoids are less differentiated than fetal organoids and native intestinal tissue. In nature, *T. gondii* sexual stages are found almost exclusively in the terminally differentiated cells in feline intestinal villi, not in the undifferentiated crypts (25). Host cell differentiation is also necessary for robust sexual development in the related Apicomplexan parasite *Cryptosporidium parvum* (2, 3). Terminally differentiated cells like absorptive enterocytes and secretory goblet cells are functionally and metabolically different from their undifferentiated precursors (26, 27). Given *T. gondii*’s remarkable ability to sense and adapt to numerous host cell environments, it would not be surprising if the parasite uses molecular cues unique to differentiated cells to initiate its sexual development program.

Nearly all of our enteroids had a cystic rather than budding phenotype (Fig. 2 to 5), which is consistent with a de-differentiated state. Just one cat 0 ileum sample developed budding structures after fibroblast withdrawal (Fig. 5), but we have not tested this sample for its ability to induce GRA11b expression in *T. gondii*. These ileum enteroids maintained their budding morphology through passage 45 when we preserved them in liquid nitrogen, suggesting that fibroblast withdrawal alone may promote differentiation in some cases. Changes in organoid medium formulation, addition of small-molecule epigenetic modifiers, and culture at an air-liquid interface are other possible tools for promoting differentiation in intestinal organoids (2, 3, 10, 28).

Feline enteroids are an important tool for studying viral and parasitic infections, but their unusually early growth arrest limits their usefulness. We showed that fibroblast feeder cells extend the lifetime of feline enteroids. Monolayers derived from fibroblast-supplemented organoids support pre-sexual development of the medically important parasite *T. gondii*. In the future, we plan to optimize our protocols to robustly support ileum and colon enteroids. We hope that replacing felines with feline enteroids will reduce the number of cats used in research, particularly for feline parasites like *T. gondii* and *Hammondia hammondi* (29) and feline coronaviruses (6), including SARS-CoV-2 (30). Our fibroblast supplementation method may also enhance the growth and survival of enteroids from other species whose intestinal tissue grows slowly or prematurely senesces.

## MATERIALS AND METHODS

### Growth and inactivation of human foreskin fibroblasts

Human foreskin fibroblasts (HFF; ATCC, SCRC-1041) were cultured in high-glucose, no-glutamine DMEM supplemented with 10% fetal bovine serum, 2 mM L-glutamine, 100 U/mL penicillin-streptomycin, and 10 mM HEPES at 37°C in a 5% CO­2_2_ atmosphere. Confluent HFFs were then treated with mitomycin C (MMC) at 10 µg/mL and incubated at 37°C for 2–3 h followed by two PBS washes to remove traces of MMC, dissociation with 20 µL/cm2^2^ of 0.025% trypsin-EDTA for 3–5 minutes, then centrifuged for 3–5 minutes at 500 × *g*. The growth-inactivated HFFs were then seeded at 200,000 cells/ cm2 ^2^ into 24-well plates and used as feeder cells for up to 8 days.

### Animals

Mice were treated in compliance with the guidelines set by the Institutional Animal Care and Use Committee (IACUC) of the University of Wisconsin School of Medicine and Public Health (protocol #M005217). Cats were euthanized for reasons unrelated to this specific research and were treated in compliance with the guidelines set by IACUC protocols.

### Organoid media

Conditioned media containing Wnt3a, R-Spondin, and Noggin were produced from the L-WRN cell line (ATCC CRL-3276) cultured in Advanced DMEM/F12, 20% fetal bovine serum, 2 mM Glutamax, 10 mM HEPES, and 100 U/mL penicillin/streptomycin, as previously described (31). L-WRN conditioned media were frozen at −20°C in 25 mL aliquots until time of use. Media for culturing enteroids contained 40% Advanced DMEM/F12, 50% L-WRN conditioned media, 2 mM GlutaMAX, 10 mM HEPES, 1× B27, 1× N2, 1× insulin/transferrin/selenium, 50 ng/mL human EGF, 10 mM nicotinamide, 2.5 µM CHIR-99021, 10 µM Y-27632, 0.5 µM A-83-01, 10 µM SB202190, 10 nM human gastrin (all components listed in Table 1), and sterile filtered. Media were kept at 4°C in the dark and used for up to 2 weeks before discarding. Infection media were identical except CHIR-99021, Y-27632, A-83-01, and SB202190 were omitted, and 200 µM linoleic acid-BSA was added.

### Linoleic acid preparation

Linoleic acid-BSA was prepared by conjugating linoleic acid oil (Nu-Chek Prep U-59-A) to fatty acid-free BSA (Sigma A8806-5G). We prepared BSA at 100 mg/mL (10% wt/vol) in PBS and filter sterilized using a SteriFlip 0.22-µm filter (EMD Millipore SCGP00525). Linoleic acid-BSA solutions were prepared by adding 2 µL linoleic acid to 1 mL of 10% wt/vol BSA plus 4 µL per mL 1M NaOH. Solutions were rotated at 37°C for ~3 h or until oil droplets were no longer visible, to obtain a 7 mM solution. Solutions were filtered through a 0.8-µm filter, aliquoted, and stored at −20°C until the time of experiments. LA was used at 200 µM, comprising ~2.9% of the total infection medium volume.

### Crypt isolation

In total, 3 cm of jejunum and ileum sections were obtained from four young cats and two mice. The section’s lumens were washed with a cold PBS solution (phosphate buffered saline, 1× penicillin/streptomycin, 25 µg/mL gentamicin), cut open, and cut into 1 cm pieces. The small sections were then washed three times by gentle rotation for 30 s in cold PBS solution. As a final wash, sections were rotated in PBS solution for 20 min at 4°C. Sections were then placed in 3 mM EDTA in PBS on wet ice for 30 min with no agitation, followed by another 30-min incubation in ice-cold 3 mM EDTA in PBS with tube rotation at 4°C. Sections were shaken vigorously for 30 s to release the crypts, inspected by light microscope, and passed through a 70-µm cell strainer to remove villi. Crypts were spun at 250 × *g* for 10 min at 4°C; the supernatant was removed and resuspended in 250–500 µL enteroid media. We recommend diluting approximately 10 µL enteroid solution to 40 µL Matrigel for plating. Dilution may need to be determined empirically; the goal is to maximize enteroid outgrowth. For plating, begin at Step 10 of the “Culturing enteroid plugs” protocol below.

### Culturing enteroid plugs

1. Enteroids should be passaged every 3–7 days or when enteroid cores become dense with necrotic cells. MMC-treated HFFs are stable for 6–8 days in enteroid media, although they decline in health either due to MMC treatment or to enteroid media. Note: If enteroids grow slowly, they can be carefully and gently moved to a new HFF monolayer by grasping their coverslip with forceps. We recommend practicing with this technique before working with valuable enteroids.
2. Visually assess enteroids to determine passage density. Either count enteroids and plan to seed 50–100 enteroids per new well for 24-well plates or passage at 1:3–1:4 as a starting point. Adjust ratios for enteroid density and expect small, budding, or dying enteroids to be less proliferative than large spherical enteroids without necrotic cores. For example, for the enteroids depicted in Fig. 3, we would recommend passaging Cat 1 Jejunum p8 HFF+ enteroids at a ratio of 1:8, 1:4 for Cat 2 Jejunum p6 HFF+, and 1:1 for the Cat 1 and Cat 2 Jejunum HFF− samples. We would pass the Cat 0 p25 Ileum HFF- sample in Fig. 5D at 1:2.
3. Preparation: Make sure HFFs in HFF plates are confluent. UV treat non-fibroblast plates for 15 min and pre-warm them at 37°C. Pre-chill a tabletop microcentrifuge to 4°C. Pre-chilled PBS with 1% FBS should be kept on wet ice. Pre-warm enteroid media to 37°C. Thaw Matrigel aliquots on wet ice. Note on glass coverslips: HFF-supplemented enteroids need to be plated on glass coverslips to prevent their direct contact with the HFF monolayer. If direct comparisons between HFF-supplemented and non-supplemented enteroids are required, we recommend adding and UV-ing glass coverslips to non-fibroblast plates for consistency. We autoclave glass coverslips and store them in a glass Petri dish with a taped lid. Other notes: In our hands, adding 1% FBS to PBS aids in separating enteroids from Matrigel, especially cat enteroids. Our protocol assumes use of 24-well plates; adjust volumes of Matrigel, enteroids, trypsin, and PBS if using other plates.
4. Beginning passage: Remove media from enteroids and place the 24-well plates on wet ice to begin dissolving Matrigel. Add 250−500 µL ice-cold PBS to each well. Dislodge plugs with pipette tip, avoiding the MMC-treated fibroblast monolayer if applicable. Aspirate and dispense the PBS-containing dislodged plugs twice to aid in dissolving Matrigel.
5. Break open enteroids and further disrupt Matrigel by slowly aspirating liquid through a 27G needle attached to a 1, 3, or 5 mL syringe, avoiding bubbles. Dispense back into the well. Repeat the aspiration step, then dispense into a 1.7 mL Eppendorf tube. Pool up to four wells of enteroids per tube to aid in pelleting. Centrifuge at 2,500 × *g* for 10–15 s at 4°C. Aspirate PBS and as much Matrigel as possible without disturbing the underlying cell pellet. Repeat PBS, spin, and remove the supernatant once more.
6. Trypsin treatment was used as necessary for mouse enteroids when enteroids were too small to lyse with a syringe. Trypsin treatments were done with 250–300 µL of 0.05%–0.25% Trypsin for 5 min at 37°C with a shake step halfway through incubation. Trypsin was inactivated with enteroid media, followed by 5–10 syringes with a 25G or 27G needle. Enteroids were pelleted at either 500 × *g* for 5 min or 2,500 × *g* for 10–15 s at 4°C and media aspirated. Note: In our hands, the 1% FBS in PBS is compatible with trypsin at the trypsin concentrations listed.
7. Resuspend enteroid pellets from step 3 or step 4 in enteroid media and spin again at 2,500 × *g* for 10–15 s at 4°C to remove residual trypsin or PBS. Remove the supernatant.
8. Resuspend enteroid pellets in enteroid media at 10 µL per enteroid plug. For example, if you pooled two high-density plugs to be split at 1:8, dilute in 160 µL enteroid media. Note: We recommend enteroid dilution at this step, not earlier. Enteroids pellet best when they are abundant.
9. Prepare an Eppendorf tube for each tissue with 10 µL enteroid solution and 40 µL Matrigel for each new well. Mix well, pipetting slowly to avoid bubbles.
10. Remove pre-warmed plates from 37°C just prior to plating. Aspirate media from HFF-containing wells and place glass coverslips directly onto HFF monolayers using sterile forceps or a Pasteur pipette attached to the vacuum apparatus. Note: To avoid shearing the monolayer, take care not to move the glass coverslip once it is placed onto the cells. We use glass coverslips because Matrigel, in direct contact with the moist HFF monolayer, spreads out rather than retaining its dome shape. Coverslips also aid in preventing HFFs from transferring from one passage to the next.
11. Add 50 µL drops of Matrigel/enteroid to the glass coverslip in each well, avoiding contact with the HFF monolayer. Note: Matrigel is viscous and must be pipetted ice cold with a pre-chilled pipette tip to evenly distribute enteroids within and across wells. Pipette slowly and do not use the second stop to avoid bubbles.
12. Quickly move enteroids to a 37°C incubator to set Matrigel until it does not jiggle when the plate is tapped (5–10 min). Note: In our hands, if Matrigel was not set quickly, cat enteroids tended to sink and attach to glass or plastic, where the spheroids opened into monolayers and died. Avoid tipping or bumping the plate.

Add 500 µL enteroid media and return to the incubator. Change enteroid media every 2–3 days.

### Enteroid enumeration and size measurements

Low-passage (passages 0–8) enteroids from cat 0 were imaged and measured at 10× magnification using an EVOS FL Auto imaging system. Photos of higher-passage cat enteroids and mouse enteroids were collected using a Zeiss Stemi 2000-C stereo microscope at 4× magnification for counting and measuring (Zeiss Zen software). Some enteroids were too small to reliably count, and Matrigel plugs that leaked beyond the edges of coverslips during plating were not counted.

### Preparing and culturing enteroid monolayers

1. Prepare a working solution of 1.0 mg/mL rat tail collagen on ice using the following volumes per 1 mL of solution: 100 µL 10× PBS, 4.6 µL 0.5 M NaOH, 795 µL sterile water, and 101 µL of 9.9 mg/mL rat tail collagen. Recommended range of working stock collagen concentrations: 0.5–2.0 mg/mL. Collagen stock concentrations vary by lot. The volume of NaOH should be adjusted to the volume of collagen added, at a ratio of 1:0.023 collagen:NaOH. Reagents should be added in the order listed.
2. Add enough collagen solution to wells to completely coat the bottoms of the wells. After even coating, remove the collagen with a pipette and transfer to the next coverslip-containing well until all coverslips are coated. Cure collagen in a 37°C humidified incubator with CO2_2_ for 3–6 h.
3. UV treat collagen-coated wells with UV for 15 min.
4. Cover collagen with cell culture media (usually HFF culturing media), then incubate overnight in a 37°C humidified incubator with CO_2_.
5. Add enteroids: Follow the “Culturing enteroid plugs” protocol above through the trypsinization step. Wash once with enteroid media and centrifuge at 2,500 × *g* for 10–15 s to remove residual trypsin. Resuspend cell pellets in enteroid media and distribute evenly among wells. Add more media immediately after cell plating for final volumes of ~ 500 µL for 24-well plates or ~1.5 mL for 12-well plates. Recommended dilution: 3–4 (24-well plate) wells of monolayers per 1 (24-well plate) well of enteroids.
6. Change enteroid media every 2–4 days. Note: Enteroid monolayer attachment and health are more sensitive to cold and desiccation than organoids in Matrigel. Use fully pre-warmed media for medium changes, perform medium changes quickly, and consider placing a warm plate between the enteroid plate and laboratory surfaces.
7. Monolayers can be plated on collagen-coated glass coverslips for imaging. Simply add coverslips prior to adding collagen, ensure the coverslip is completely coated, and press the coverslip to the bottom of the plate using a sterile pipette tip. If cell attachment is poor, consider other attachment matrices such as gelatin, entactin-collagen-laminin (ECL), or 10% Matrigel. Also, consider poly-L-lysine-coated coverslips.
  - Cat monolayers were prepared using ECL and glass coverslips. ECL was added to wells of 4-well plates at 10 µg/cm2^2^ diluted in advanced DMEM/F12. ECL was incubated either 1 h at 37°C or overnight at 4°C and removed just prior to the addition of the cell solution.
  - Mouse monolayers were prepared using collagen and poly-L-lysine-coated coverslips (Electron Microscopy Sciences 72292-02).

### Parasites

Oocysts from the *T. gondii* type II ME49 ∆hpt luciferase and TgShSp1 strains were produced as previously described (32, 33) and stored in a 2% sulfuric acid solution at 4°C until the time of infection. The age of these oocysts at the time of infection was approximately 2 years.

### Animal infections for parasite propagation

Male C57BL/6 mice were used for all animal infections. ME49 ∆hpt luciferase oocysts were mixed well and diluted to 1.25 × 10^4^/mL in 5 mM HEPES and PBS. TgShSp1 oocysts were mixed well and diluted to 5 × 10^3^/ mL in 5 mM HEPES and PBS. Mice were injected intraperitoneally with 200 µL oocyst solution for doses of 2.5 × 10^3^ and 1 × 10^3^ per mouse for ME49 and TgShSp1, respectively. Mice were monitored daily for symptoms of toxoplasmosis and euthanized if they reached endpoint criteria. Approximately 90% of animals infected with ME49 and 100% of animals infected with TgShSp1 survived until euthanasia for brain cyst collection at 28–90 days post-infection.

### Organoid infections

Cat and mouse monolayers were grown 1–4 weeks prior to infection in 4-well plates on top of glass coverslips pre-coated with ECL (cats) or collagen (mice). At 24 h prior to infection (day −1), organoid media were changed to infection media. See “Organoid media,” above, for more details on infection medium composition. On the day of infection (day 0), mice were euthanized, and brain cysts were purified using a Percoll gradient. Cysts were digested for 30 min at 37 ˚C in pepsin-acid solution: 0.01 mg/mL pepsin (Thermo Scientific AAJ6167909) in 1% NaCl, pH 2.1, spun, and resuspended in infection media. Free bradyzoites were counted on a hemocytometer and added to monolayers at 20,000 bradyzoites per 1.9 cm2^2^. The media were changed at 2 days post-infection.

### Immunofluorescence assays

At 5 days post-infection, infected monolayers were fixed with 4% paraformaldehyde, permeabilized for 5 min with 0.2% Triton-X-100, and blocked for 1 h at room temperature or overnight at 4°C in 3% BSA. Primary antibodies were incubated in 3% BSA overnight at 4°C. Antibodies used for *T. gondii* were 1:1,500 rabbit polyclonal anti-*T*. *gondii* (Thermo Scientific PA17252) with 1:1,000 goat anti-rabbit Alexa 594 secondary antibody (Thermo Scientific A-11005). Antibodies used for GRA11b staining were 1:750 monoclonal mouse anti-GRA11b, with 1:500 goat anti-mouse Alexa 488 secondary antibody (Thermo Scientific A-11001). Secondary antibodies and DAPI were incubated together for 1 h at RT. Coverslips were mounted onto slides and imaged on a Zeiss LSM 800 Laser Scanning confocal microscope. Values were imported into Excel and then R for statistical analysis and figure preparation.

### Statistical analysis

Figure preparation and statistical analyses were performed in RStudio using the R programming language.
